# Exercise mitigates a gut microbiota-mediated reduction in adult hippocampal neurogenesis and associated behaviours in rats

**DOI:** 10.1038/s41398-024-02904-0

**Published:** 2024-04-24

**Authors:** Sarah Nicolas, Sebastian Dohm-Hansen, Aonghus Lavelle, Thomaz F. S. Bastiaanssen, Jane A. English, John F. Cryan, Yvonne M. Nolan

**Affiliations:** 1 Department of Anatomy and Neuroscience, University College Cork, Cork, Ireland; 2 APC Microbiome Ireland, University College Cork, Cork, Ireland; 3 INFANT Research Centre, Cork University Hospital, Wilton, Cork, Ireland

**Keywords:** Hippocampus, Physiology

## Abstract

Lifestyle factors, especially exercise, impact the manifestation and progression of psychiatric and neurodegenerative disorders such as depression and Alzheimer’s disease, mediated by changes in hippocampal neuroplasticity. The beneficial effects of exercise may be due to its promotion of adult hippocampal neurogenesis (AHN). Gut microbiota has also been showed to be altered in a variety of brain disorders, and disturbances of the microbiota have resulted in alterations in brain and behaviour. However, whether exercise can counteract the negative effects of altered gut microbiota on brain function remains under explored. To this end, chronic disruption of the gut microbiota was achieved using an antibiotic cocktail in rats that were sedentary or allowed voluntary access to running wheels. Sedentary rats with disrupted microbiota displayed impaired performance in hippocampal neurogenesis-dependent tasks: the modified spontaneous location recognition task and the novelty suppressed feeding test. Performance in the elevated plus maze was also impaired due to antibiotics treatment. These behaviours, and an antibiotics-induced reduction in AHN were attenuated by voluntary exercise. The effects were independent of changes in the hippocampal metabolome but were paralleled by caecal metabolomic changes. Taken together these data highlight the importance of the gut microbiota in AHN-dependent behaviours and demonstrate the power of lifestyle factors such as voluntary exercise to attenuate these changes.

## Introduction

The gut microbiota comprises the trillions of microorganisms inhabiting the gastrointestinal tract and represents an important component in the bidirectional communication between the brain and the gut. Numerous studies have now implicated the gut microbiota in the aetiology of neurological and neuropsychiatric diseases [1] including Alzheimer’s [2], Parkinson’s [3] and depression [4]. The gut microbiota composition changes throughout the lifespan [5], depending on environmental or lifestyle factors, notably diet and exercise [6–8].

A growing body of evidence shows that exercise changes the gut microbial composition [9, 10] and related metabolites, that those changes confer benefits on health and may delay disease progression [11]. For example, it has been shown that exercise impacts gut microbiota diversity and increases taxa that produce health-promoting metabolites such as short chain fatty acids (SCFA) [12]. A sedentary lifestyle represents a risk factor for cognitive impairments [13], and many studies now report that physical activity has beneficial effect on brain health [14, 15]. Exercise increases level of growth factors such as brain-derived neurotrophic factor (BDNF) [16], vascular endothelial growth factor [17] or insulin growth factor-1 (IGF-1) [18] in the brain. Interestingly, the gut microbiota has been shown to modulate some of these factors [19]. Thus, the ability of exercise to shape the microbiome and subsequent gut-brain-associated pathways may have crucial implications for the development of strategies to treat neurological and psychiatric disorders.

The main brain structure affected by exercise is the hippocampus [20], which plays a role in learning, memory, and in mood-related behaviours. The hippocampus is also capable of generating new neurons across the lifespan in a process called adult hippocampal neurogenesis (AHN) [21]. This form of brain plasticity is involved in cognitive tasks which require spatial and contextual memory [22] and in anxiety-related behaviours [23]. There is consensus that AHN is predominantly required for pattern separation [24, 25], which is the ability to discriminate between memories of similar experiences or environments. Notably, rodent studies have shown that exercise is a robust enhancer of certain types of cognitive function through its ability to increase AHN [26, 27].

While the gut microbiome regulates cognitive behaviours [7, 28, 29], neuroplasticity [30, 31] and AHN [4, 32, 33], the interaction between the gut microbiota and exercise on AHN and associated function remains unresolved. In this study, we examined the ability of exercise to ameliorate the potential negative effects of gut microbiota disruption using a cocktail of antibiotics on AHN, cognitive function, and mood related behaviours in adult male rats.

## Methods

### Animals and experimental design

**Fig. 1:**
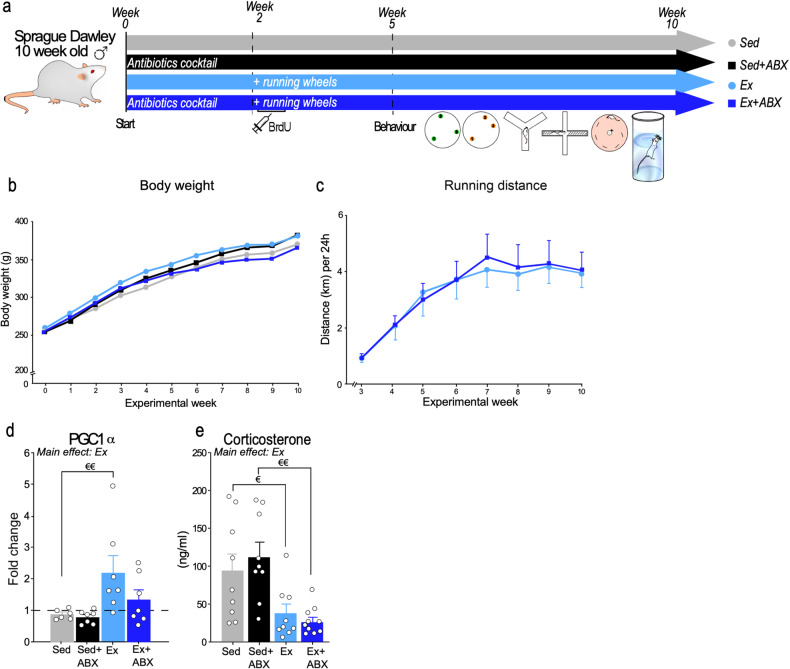
Timeline, body weight, running distance and physiological parameters. **a** Experimental timeline. All rats were pair housed and received antibiotics (ABX) in drinking water or not throughout the study. Two weeks after the beginning of ABX treatment rats were separated in 4 experimental groups, sedentary (Sed), sedentary with Abx (Sed+ABX) or had continuous access to a running wheel (Ex) or exercise with ABX (Ex+ABX). All rats received BrdU (150 mg/kg/day for 5 days) and behavioural testing commenced after 3 weeks of exercise. Exercise continued throughout testing for a total of 8 weeks. **b** Body weight of rats and (**c**) Running wheel activity (average km/24 h per rat) (*n* = 5; Repeated Measures ANOVA; main effect of time [F_(7, 63)_ = 20.38; *p* < 0.0001]) throughout the experiment. **d** Relative mRNA expression of PGC1α in skeletal muscle (*n* = 6–7; two-way ANOVA; main effect of exercise; a priori comparisons €€ *p* < 0.01). **e** Corticosterone levels (ng/ml) in plasma (*n* = 9–10; two-way ANOVA; main effect of exercise: € *p* < 0.05 and €€ *p* < 0.01). Data are graphed as means + SEM. See supplementary Table 4 for details of statistical analysis.

Adult (9 week old) male Sprague‐Dawley rats were purchased from Envigo, UK, and maintained on a 12‐h:12‐h light:dark cycle (lights on at 07:00 h) at 22 ± 1 °C, with access to standard chow (Teklad 2018S) and water *ad libitum*. Rats were pair-housed and randomly assigned to an experimental group as follows: sedentary control “Sed”, sedentary and antibiotics cocktail “Sed+ABX”, voluntary running “Ex”, and voluntary running and antibiotics cocktail “Ex+ABX” (*n* = 10; Fig. 1a). Antibiotics were administered in the drinking water and bottles were changed every second day. Rats were pair-housed in either standard housing cages (Cage size 560 × 380 × 170 mm, with hollow paper tubes and shredded paper) or in cages with continuous and free access to running wheels (Cage size 425 × 266 × 185 mm; Activity wheel 33 cm Ø, Tecniplast, UK) for the duration of the experiment. Animals were pair-housed in order to avoid any potential social isolation stress effects of single housing on hippocampal neurogenesis [34, 35]. We have previously shown that social isolation impacts upon exercise-induced changes in neurogenesis in mice [36]. Rats were placed in cages with running wheels 2 weeks after the start of antibiotic administration and were intraperitoneally injected with 5-Bromo-2’-deoxyuridine (BrdU; 150 mg/kg of body weight; #B5002, Sigma) once a day for five consecutive days. Wheel revolutions were continuously monitored and were consistent across running groups (Fig. 1c). All animal procedures were performed under authorisations issued by the Health Products Regulatory Authority (HPRA, Ireland), in accordance with the European Communities Council Directive (2010/63/EU) and approved by the Animal Experimentation Ethics Committee of University College Cork.

### Behaviours

All rats underwent behavioural testing (Pattern separation; Y-maze; Novelty suppressed feeding; Elevated plus maze (EPM) and Forced swim test) starting three weeks following initiation of exercise and BrdU injection. Rats were singly housed and acclimated to the testing room at least 45 min prior to each test. All behavioural tests were conducted during the light phase (8:30 am to 5:30 pm). All apparatus and objects were cleaned with a 70% ethanol solution between exposures of each animal to remove odour cues. Where possible, behaviours were scored in a blinded fashion or by using automated Ethovision video tracking software (XT 7.0; Noldus, The Netherlands) and AnyMaze (Stoelting, Europe). See *SI Materials and Methods* for details.

### Tissue collection and post-mortem analysis

At week 10, five rats per group were euthanized with an injection of pentobarbital (90 mg/kg; i.p.), blood was collected by cardiac puncture, gut and muscle was dissected, and rats were transcardially perfused with saline followed by 4% (v/v) paraformaldehyde (#30525–89–4; Fisher) for immunohistochemical analysis. Brains were postfixed, cryoprotected in 30% sucrose and subsequently flash frozen. Brains were coronally sectioned (40 µm) in a 1:12 series through the hippocampus and preserved in an anti-freeze solution at –20 °C before processing (see *supplementary information*). The remaining five rats per group were euthanized by rapid decapitation. Hippocampus, colon, caecum content, and muscle were dissected and immediately snap frozen in liquid nitrogen. Blood samples were collected into ethylene-diamine tetra-acetic acid (EDTA) tubes and centrifuged (15 min, 5000 rpm). The resulting plasma was stored at −80 °C until further analysis (see *supplementary information for details*).

### Metabolomics analysis

Metabolomics screening was performed on caecum content and hippocampus by MS-OMICS (Denmark) as previously described (https://www.msomics.com/metabolomics-methods/technical-information/). See *SI Materials and Methods*.

### Statistical analysis

All datasets were assessed for normal distribution and equality of variances using Shapiro-Wilk test. Datasets that were normally distributed were statistically compared using two-way ANOVA followed by Tukey’s multiple comparison test. Non-parametric datasets were statistically compared using Kruskal–Wallis test followed by *post-hoc* Dunn’s test. A *p* value of less than 0.05 was considered significant. For metabolomics differential expression analysis, metabolite features were considered statistically significant at a false discovery rate (FDR)-corrected *p* < 0.05. For Spearman correlation analyses of metabolites and biochemical, immunohistochemical, and behavioural data, results were considered nominally significant at *p* < 0.05, and FDR-based correction for multiple comparisons was applied, with a significance threshold of 5%. Details of all statistical analysis are in Supplementary Tables 4–8.

## Results

### Gut microbiota disruption did not change body weight and running activity

Neither voluntary exercise nor antibiotics treatment affected rat body weight (Fig. 1b). This is similar to previous reports showing that rats that start to exercise in adulthood did not lose weight when compared to their sedentary counterparts [37, 38], and that voluntary running-induced body weight loss is mainly observed when exercise is initiated during adolescence [27]. The average distance run over the course of the experiment was comparable to previous published reports [39, 40] and similar in the exercise (Ex) and exercise+antibiotics (Ex+ABX) group (Fig. 1c). Proliferator-activated receptor gamma coactivator-1a (PGC1a), which has previously been shown to mediate an exercise-induced increase in hippocampal BDNF [41] was significantly increased in skeletal muscle by exercise (Fig. 1d). It has been previously shown that the lack of gut microbiota in germ-free mice increased the corticosterone response to stress [42], while voluntary exercise reduced the corticosterone response to stress [43], suggesting that both can regulate the HPA axis. Two-way ANOVA revealed that exercise decreased plasma corticosterone but there was no effect of antibiotics nor the exercise x antibiotics interaction. Rats that exercised had lower level of plasma corticosterone compared to their sedentary control regardless of antibiotics treatment (Sed vs Ex *p* = 0.0327; Sed+ABX vs Ex+ABX *p* = 0.0084) (Fig. 1e).

### Gut microbiota disruption-induced low-grade peripheral inflammation and changes in gut physiology

**Fig. 2:**
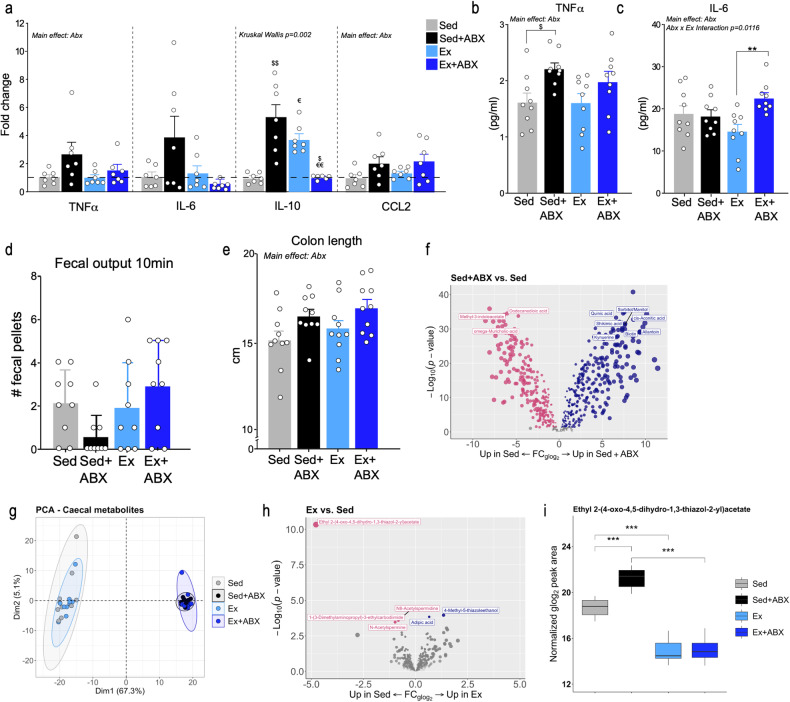
Peripheral inflammatory measures and metabolomic analysis of caecal content. Effect of antibiotics and exercise on (**a**) relative mRNA expression of TNFɑ (*n* = 7; two-way ANOVA; main effect of Abx), IL-6 (*n* = 7; Kruskal–Wallis test), IL-10 (*n* = 7; Kruskal–Wallis test; *p* = 0,0002; Abx effect: ^$$^*p* < 0.01; Exercise effect: ^€^*p* < 0.05), and CCL2 (*n* = 7; two-way ANOVA; main effect of Abx) in the colon. Plasma concentration of (**b**) TNFɑ (pg/ml) and (**c**) IL-6 (pg/ml) (*n* = 9; two-way ANOVA; main effect of Abx: a priori comparisons ^$^*p* < 0.05; interaction Abx x Ex **p < 0.01). **d** fecal output (*n* = 9; Kruskal–Wallis test) (**e**) colon length at the end of the study (*n* = 10; two-way ANOVA; main effect of Abx); **f** Volcano plot of caecal metabolites for Sed+ABX and Sed rats. Top ten most significant metabolites are highlighted. **g** Principal Component Analysis (PCA) of caecal metabolomics (**h**) Volcano plot of caecal metabolites for Sed and Ex rats. **i** Normalised peak areas for the metabolite Ethyl 2-(4-oxo-4,5-dihydro-1,3-thiazol-2-yl)acetate; ***p < 0.001 (FDR-adjusted; Limma *p* value). Ex vs Sed: glog2 fold change = −4.8, *p*_FDR-adjusted_ = 2.8E-8; Ex+ABX vs Sed+ABX: glog2 fold change = −6.2, *p*_FDR-adjusted_ = 1.9E-11; Sed+ABX vs Sed comparison glog2 fold change = 2.6, *p*_FDR-adjusted_ = 3.2E-5. Data are graphed as means + SEM. Abbreviation: FC fold change, glog_2_ generalised logarithm base 2. See Supplementary Table 5 for details of statistical analysis.

There was a significant main effect of antibiotics on colonic TNFα mRNA expression while colonic IL-6 mRNA was not significantly affected by either intervention. We found that exercise and antibiotics independently increased the mRNA expression of IL-10 (*p* = 0.0002, Kruskal–Wallis), while the combination of both significantly reduced its expression. Two-way ANOVA showed a main effect of antibiotics on the expression of the chemokine CCL2 in the colon while neither exercise nor the exercise x antibiotics interaction affected its expression (Fig. 2a). We next assessed if the modest increase in gut inflammation was reflected in the blood. Two-way ANOVA analysis of plasma levels of inflammatory cytokines showed only a main effect of antibiotics on the concentration of TNFα. Subsequent planned comparison showed that antibiotics increased levels of TNFα in sedentary rats only (Sed vs Sed+ABX *p* = 0.0475) (Fig. 2b). Similarly, IL-6 levels were not affected by exercise but by antibiotics and by the exercise x antibiotics interaction. *Post hoc* analysis revealed that antibiotics increased IL-6 plasma level only in the exercise group (Ex vs Ex+ABX *p* = 0.0063) (Fig. 2c). We observed that there was a trend towards an effect of antibiotics on faecal output (*p* = 0.0609, Kurskal–Wallis; Fig. 2d). There was a main effect of antibiotics on the colon length (Fig. 2e), and caecum weight (Fig. S1a, b).

### Gut microbiota disruption impacted the caecal but not hippocampal metabolome

Because the long-term disruption of gut microbiota induced physiological changes in the gut, we next performed mass spectrometry-based untargeted metabolomics analysis of caecal content and hippocampal tissue (See *SI Materials and Methods*). The antibiotic treatment caused a large shift in the caecal metabolome (Fig. 2f–i**;** Fig S1**;** Fig S2). Of the 610 caecal metabolites quantified, 527 were differentially regulated (*p*_FDR-adjusted_ < 0.05) between Sed+ABX and Sed rats (Fig. 2f, g). Pathway analysis on differentially expressed metabolites revealed an enrichment in aminoacyl-tRNA synthesis (18 metabolites implicated), the citric acid cycle (7 metabolites implicated), and several amino acid-related pathways (Supplementary Table 3). Conversely, exercise had few effects on the caecal metabolome, as only six metabolites were differentially regulated between Ex and Sed rats (Fig. 2h) and only one in the Ex+ABX vs Sed+ABX comparison (Fig. S1b). The metabolite ethyl 2-(4-oxo-4,5-dihydro-1,3-thiazol-2-yl)acetate was consistently downregulated by exercise (Sed vs Ex: *p*_FDR-adjusted_ = 2.8E-8; ABX vs ABX+Ex *p*_FDR-adjusted_ = 1.9E-11) but was also increased by antibiotics in the Sed vs Abx comparison (*p*_FDR-adjusted_ = 3.2E-5; Fig. 2i). Of the 210 hippocampal metabolites quantified and annotated, two were differentially abundant as a function of antibiotics treatment in the Sed+ABX vs Sed comparison (Fig. S2a, b). The metabolite trimethylamine-N-oxide (TMAO) was decreased between Sed+ABX and Sed rats (*p*_FDR-adjusted_ = 1.5E-5) and below the limit of the detection in Ex+ABX rats (Fig. S2e). This is consistent with the gut microbiota’s role in the biosynthesis of TMAO [44]. Interestingly, ergothioneine, a precursor of TMAO, was identified as significantly increased in the hippocampus (*p*_FDR-adjusted_ = 0.045) following antibiotic treatment (Fig. S2b–g), but was not detected in caecal content. Several other TMAO precursors, such as carnitine metabolites and choline, were altered in the caecum, but not the hippocampus (Fig. S1c S2g**;** Supplementary Table 2), in the Sed+ABX vs Sed comparison. The metabolite ethyl 2-(4-oxo-4,5-dihydro-1,3-thiazol-2-yl)acetate was not differentially expressed in the hippocampus (all comparisons, *p* > 0.05; Fig S2f). These results suggest loss of TMAO in the hippocampus by antibiotics-induced disruption of gut bacteria. No metabolites were differentially regulated as a function of exercise in the hippocampus in the Ex vs Sed and Ex+ABX vs Sed+ABX comparisons (all *p*_FDR-adjusted_ > _0.05_); though, 9 and 17 features, respectively, reached nominal significance (*p* < 0.05).

### Exercise prevented a gut microbiota disruption-induced decrease in pattern separation

**Fig. 3:**
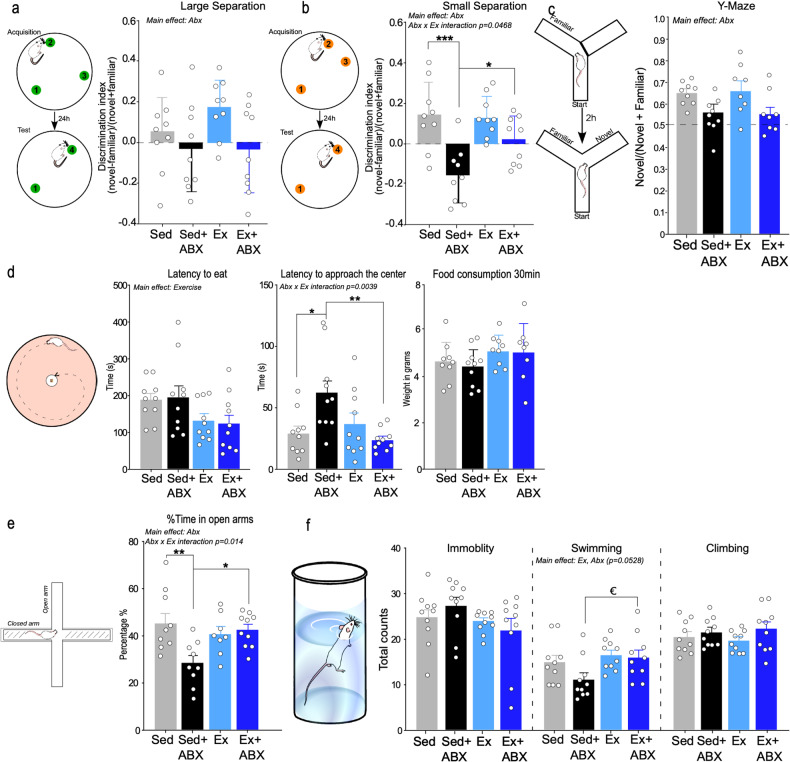
Effect of antibiotics and exercise on cognitive, anxiety- and despair-like behaviours. **a** Schematic representation and discrimination ratio in the large separation task (*n* = 9; two-way ANOVA; main effect of Abx) and (**b**) in the small separation task (pattern separation) (*n* = 9; two-way ANOVA; interaction Abx x Ex; *p < 0.05;***p < 0.001); **c** Schematic representation and discrimination ratio between the exploration of the novel vs familiar arm during the first 2.5 min of the 2-trial Y-Maze test (*n* = 8–9; two-way ANOVA; main effect of Abx); **d** Schematic representation; latency to eat (*n* = 10; two-way ANOVA; main effect of Ex) and latency to first approach the centre (*n* = 10; two-way ANOVA; interaction Abx x Ex; *p < 0.05;**p < 0.01) in the novelty suppressed feeding test (NSFT) ; **e** Schematic representation and % of time spent in the open arms of the elevated plus maze (EPM) (*n* = 8–10; two-way ANOVA; interaction Abx x Ex; *p < 0.05;**p < 0.01); **f** Schematic representation and immobility, swimming (*n* = 10; two-way ANOVA; main effect Ex; a priori comparisons ^€^*p* < 0.05) and climbing score in the Forced Swim Test (FST). Data are graphed as means + SEM. See Supplementary Table 6 for details of statistical analysis.

Changes in gut inflammation and the gut metabolome can impact upon hippocampal-dependent cognitive behaviours [45, 46] thus, given our observations, and the established exercise-induced enhancement of hippocampal function [47, 48] we examined the effects of exercise and antibiotics on hippocampal-dependent memory. Two-way ANOVA revealed that antibiotics but not exercise nor their interaction affected rats performance in the large separation paradigm of the MSLRT, a task of location discrimination in conditions of low contextual overlap (Fig. 3a). In the small separation, a condition of high contextual overlap (pattern separation) that requires AHN [24, 49], two-way ANOVA revealed a trend toward the effect of exercise on rats ability to pattern separate [*p* = 0.0742]. Both antibiotics and the interaction exercise x antibiotics significantly affected rats performance in this task (Fig. 3b). Subsequent *post hoc* analysis showed that antibiotics decreased rats performance in the small separation task (Sed vs Sed+ABX; *p* = 0.0003) and that exercise reversed this impairment (Sed+ABX vs Ex+ABX *p* = 0.045). In the two-trial Y-maze, a test of spatial reference memory, two-way ANOVA revealed no effect of exercise or exercise x antibiotics interaction but a main effect of antibiotics on the discrimination ratio (Fig. 3c), which was independent of rats’ locomotor activity in both trials on the Y-maze (Supplementary Fig. 3a).

### Exercise prevented gut microbiota disruption-induced changes in anxiety-like behaviours

Because the gut microbiota is involved in the regulation of mood-related behaviours [7, 28]. We assessed anxiety-like and despair-like behaviours in the novelty suppressed feeding test (NSFT), elevated plus maze (EPM) and Forced Swim test (FST; Fig. 3). A decreased latency to eat in the NSFT has been associated with increased AHN induced by antidepressant drugs [50, 51]. Two-way ANOVA revealed a main effect of exercise on the latency to eat (Fig. 3d). However, we cannot rule out that results from rats who exercised may have been driven by hunger as we found a significant weight loss induced by exercise **(**Fig S3b) as well as a significant correlation between the latency to eat and % body weight loss in rats in the exercise group, but not in rats in the sedentary, antibiotics nor exercise+antibiotics groups (Fig. S3c). The latency to approach the centre was significantly affected by the interaction between antibiotics and exercise. *Post hoc* analysis showed that there was an increased latency to reach the centre in antibiotics-treated rats (Sed vs Sed+ABX *p* = 0.0192), which was attenuated in rats that were also exposed to exercise (Sed+ABX vs Ex+ABX *p* = 0.0051). This result is independent of body weight loss as no significant correlations were observed between these parameters in all experimental groups (Fig. 3d). In addition, there was no difference in food consumption 30 min post-test (Fig. 3d). In the EPM (Fig. 3e), two-way ANOVA showed that antibiotics and the antibiotics x exercise interaction had an overall effect on percentage of time spent in the open arms. *Post hoc* analysis showed that antibiotics-treated rats spent less time in the open arms (Sed vs Sed+ABX *p* = 0.0096), indicative of an anxiogenic effect, which was attenuated in rats that were also exposed to exercise (Sed+ABX vs Ex+ABX *p* = 0.028) (Fig. 3e). Finally, we assessed despair-like behaviour in the FST (Fig. 3f). Climbing and immobility behaviours were unaffected by either exercise or antibiotics. Swimming behaviour was significantly affected by exercise. Antibiotics-treated rats who were exposed to exercise swam more than sedentary antibiotics-treated rats (Sed+ABX vs Ex+ABX *p* = 0.0212) (Fig. 3f). Overall, the data suggest that antibiotics increased anxiety-like behaviour and induce a slight enhancement in depressive-like behaviour which was attenuated by exercise.

### Exercise mitigated the negative impact of gut microbiota disruption on adult hippocampal neurogenesis

**Fig. 4:**
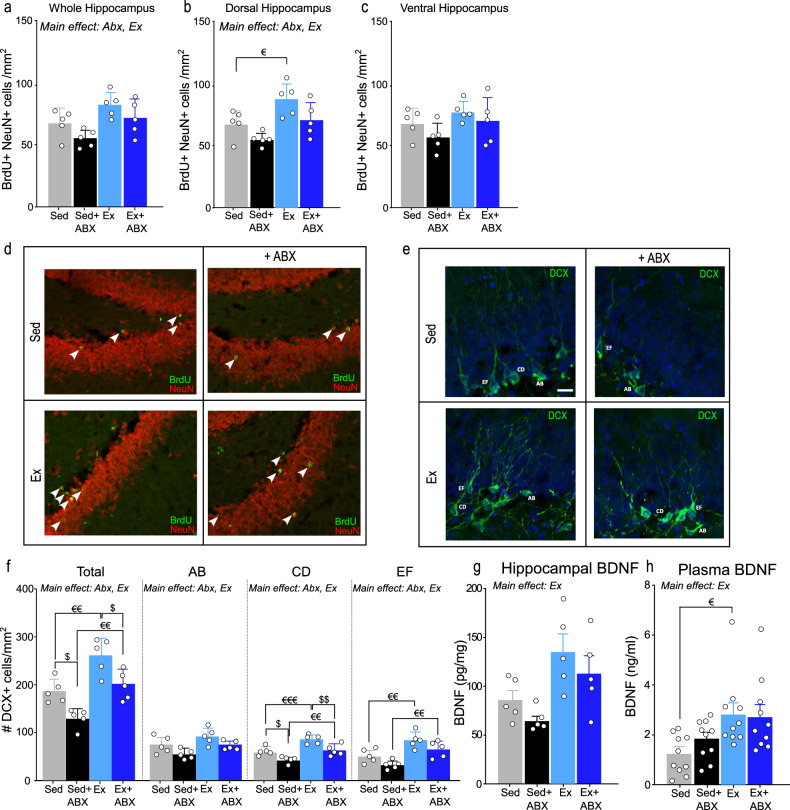
Hippocampal neurogenesis and BDNF levels. Immunohistochemical measurement of the survival of new neurons (BrdU and NeuN positive cells) in brain sections through (**a**) the whole (*n* = 5; two-way ANOVA; main effect of Abx; main effect of Ex) (**b**) dorsal (*n* = 5; two-way ANOVA; main effect of Abx; main effect of exercise; a priori comparisons ^€^*p* < 0.05) and (**c**) ventral hippocampus (*n* = 5; two-way ANOVA). **d** representative images of BrdU/NeuN at X20 magnification (*n* = 5). **e** representative images of DCX+ cells at X40 magnification (**f**) DCX counts following classification based on their dendritic tree morphology and total count of all DCX+ cells (*n* = 5; two-way ANOVA; main effect of exercise: a priori comparisons ^€€^*p* < 0.01; ^€€€^*p* < 0.001 and main effect of Abx; a priori comparisons ^$^*p* < 0.05; ^$$^*p* < 0.01). **g** BDNF in hippocampus (pg/mg tissue) (*n* = 5; two-way ANOVA; main effect of exercise) (**h**) BDNF (ng/ml) in plasma (*n* = 10; two-way ANOVA; main effect of exercise: a priori comparisons ^€^*p* < 0.05). Data are graphed as means + SEM. See Supplementary Table 7 for details of statistical analysis.

Previous studies have demonstrated that pattern separation requires AHN [24, 49] and that AHN can be regulated by both exercise and the microbiome [32, 47]. We assessed AHN by determining the number BrdU + /NeuN+ cells in the dentate gyrus (DG) of the hippocampus (Fig. 4a–d). Two-way ANOVA revealed a main effect of exercise and antibiotics on AHN in the whole hippocampus, but not their interaction (Fig. 4a). Subdivision of regions across the longitudinal axis revealed that again, only exercise and antibiotics alone affected AHN in the dorsal region. Planned comparison revealed that the pro-neurogenic effect of exercise was specific to the dorsal region (Sed vs Ex *p* = 0.05) (Fig. 4b). In the ventral hippocampus, neither antibiotics or exercise significantly affected AHN (Fig. 4c). In line with these findings, we observed that there was no signification interaction between antibiotics and exercise on the total number of DCX-positive cells in the DG (Fig. 4e, f). However, both factors independently affected the number of DCX+ cells such that antibiotics decreased and exercise increased the number of DCX-positive cells in the DG (Fig. 4f, left panel). We next categorised the DCX+ cells as described by Plümpe and colleagues [52]; AB were cells with very short or no processes, CD cells had intermediate processes and immature morphology, and EF cells were more mature with multi-branched dendrites (Fig. 4e). Two-way ANOVA revealed that antibiotics had a main effect on the three categories of developing neurons. Similarly, exercise had a main effect on the maturation status of neurons but there was no significant interaction between Abx and Ex. *Post hoc* analysis showed that the antibiotics-induced reduction in DCX-positive cells was specific to CD-type cells (Sed vs Sed+Abx *p* = 0.03; Ex vs Ex+Abx *p* = 0.002), while the exercise-induced increase was limited to CD-type (Sed vs Ex *p* < 0.001 ; Abx vs Ex+Abx *p* = 0.009) and EF-type (Sed vs Ex *p* = 0.0096; Abx vs Ex+Abx *p* = 0.0091) cells. (Fig. 4e). Because BDNF mediates the neurogenic effect of exercise [53] we measured BDNF in hippocampus and plasma and found that exercise significantly increased concentrations of BDNF in both hippocampus (Fig. 4g) and plasma (Fig. 4h) regardless of the antibiotics intervention. Subsequent *post hoc* test showed that rats that exercised had higher levels of plasma BDNF than their sedentary counterparts (Sed vs Ex *p* = 0.0226; Fig. 4h). Because microglia influence the neurogenic niche [54, 55], and antibiotics activate microglia in mice [56], we assessed microglia status in the hippocampus of rats exposed to exercise and antibiotics (Fig S4). Our results show that long-term antibiotics trigger a low-grade inflammatory state in the hippocampus that is not fully restored by exercise. (See supplementary information and Fig S3). Overall, these results suggest that while long-term microbiota disruption induced low grade inflammation but did not impact upon BDNF, exercise attenuated the negative impact of microbiota disruption on AHN.

### The metabolite ethyl 2-(4-oxo-4,5-dihydro-1,3-thiazol-2-yl) acetate correlates with physiological and behavioural changes induced by gut microbiota disruption

**Fig. 5:**
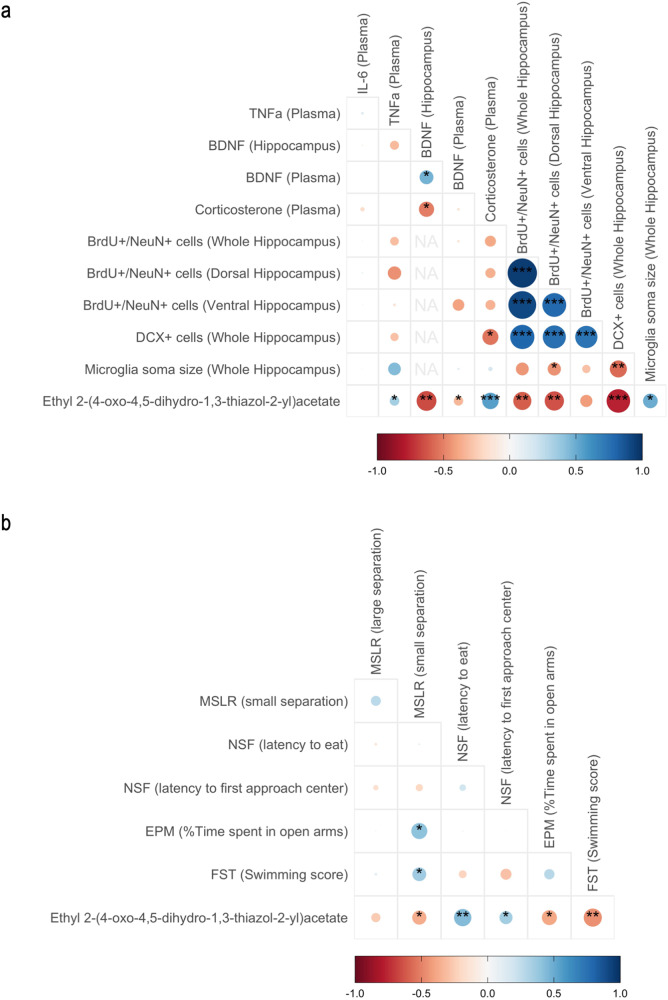
Correlation of caecal metabolite ethyl 2-(4-oxo-4,5-dihydro-1,3-thiazol-2-yl)acetate with biological and behavioural measures. **a** Spearman correlation heatmap for caecal ethyl 2-(4-oxo-4,5-dihydro-1,3-thiazol-2-yl)acetate normalised expression, plasma BDNF, IL-6, TNFa, corticosterone, hippocampal BDNF protein levels, number of BrdU^+^/NeuN^+^ and DCX^+^ cells per mm^2^ in the whole, dorsal, and ventral hippocampus, and microglia soma size in the whole hippocampus. **b** Spearman correlation heatmap for caecal ethyl 2-(4-oxo-4,5-dihydro-1,3-thiazol-2-yl)acetate normalised expression, performance in the MSLR (large and small separation), NSF, EPM and FST (swimming behaviour); *p < 0.5, **p < 0.01, ***p < 0.001; unadjusted.

After adjusting for multiple comparisons, the expression level of ethyl 2-(4-oxo-4,5-dihydro-1,3-thiazol-2-yl)acetate was positively correlated with antibiotics-induced increase in plasma corticosterone (*r*_spearman_ = 0.55, *p*_FDR-adjusted_ = 0.0025) and antibiotics-induced increase of anxiety-like behaviour in the NSF (Latency to eat; *r*_spearman_ = 0.44, *p*_FDR-adjusted_ = 0.050). Interestingly, this metabolite was negatively correlated with hippocampal BNDF protein levels (*r*_spearman_ = −0.66, *p*_FDR-adjusted_ = 0.012), the number of surviving BrdU + /NeuN+ cells in the whole (*r*_spearman_ = −0.60, *p*_FDR-adjusted_ = 0.021) and dorsal (*r*_spearman_ = −0.64, *p*_FDR-adjusted_ = 0.018) hippocampus, the number of DCX+ cells in the whole hippocampus (*r*_spearman_ = −0.77, *p*_FDR-adjusted_ = 0.00061), and the swimming score in the FST (*r*_spearman_ = −0.46, *p*_FDR-adjusted_ = 0.050). We also found positive nominal correlation of the metabolite with plasma TNFa (*r*_spearman_ = 0.34, *p* = 0.043), microglial soma size (*r*_spearman_ = 0.50, *p* = 0.026) and antibiotics-induced anxiety-like behaviour in the NSF (latency to first approach to centre; *r*_spearman_ = 0.34, *p* = 0.030), and in the EPM (time spent in the open arms; *r*_spearman_ = −0.39, *p* = 0.021). In addition, ethyl 2-(4-oxo-4,5-dihydro-1,3-thiazol-2-yl) acetate was negatively correlated with plasma BDNF (*r*_spearman_ = −0.33, *p* = 0.036) and pattern separation performance in the small separation MSLR test (*r*_spearman_ = −0.37, *p* = 0.028) (Fig. 5a, b).

## Discussion

This study shows that selective peripheral and central changes induced by long term disruption of gut microbiota were partially reversed by exercise in adult male rats. We observed that antibiotic treatment led to impairments in pattern separation and anxiety-like behaviours, coupled with a decrease in AHN. We show that antibiotics induced low grade inflammation in the hippocampus by increasing the number of activated microglia, which could be linked to the decreased AHN. Increased low grade neuroinflammation was concomitant with an increase in peripheral and colonic inflammation. Moreover, we found that antibiotics but not exercise induced profound changes in the caecal metabolome and that these were mostly not reflected in the hippocampal metabolome. Exercise partially reversed the anxiety-like behaviours and pattern separation impairment induced by antibiotics by maintaining AHN and BDNF levels and reducing the activation state of the microglia and peripheral inflammatory status.

The association between the gut microbiota and peripheral inflammation has been already established [57, 58]. Microbiota manipulation with fecal microbiota transplant (FMT) regulates inflammation in the colon and plasma in male mice [59]. Here, we found that antibiotics increased plasma levels and colonic expression of TNF-α and that exercise prevented these changes. These results reflect previous findings showing that exercise reduced colonic inflammation induced by a high fat diet in male mice [60], which is known to affect gut microbiota composition [61]. IL-6 signalling is known to be altered by exercise [62] and the microbiome [63]. Here we found that while exercise alone maintained low level of IL-6, the combination of exercise and antibiotics induced an increase in plasma levels of IL-6 while the changes in colonic expression of IL-6 mRNA induced by antibiotics and exercise were more subtle. In addition, IL-6 can regulate the expression of anti-inflammatory cytokines such as IL-10 in the colon [64], which is in line with what we observed.

As a result of long-term microbiota disruption, the number and soma size of microglial cells was increased in both sedentary and exercised male rats. Elegant mixed-gender studies in mice have confirmed that the gut microbiota regulates microglia development and maturation [65]. Antibiotics administration to male mice increased microglia size and number [56] supporting the observations in our study. Another study found that neither antibiotics nor exercise affected microglia status in the whole brain but impacted Ly6C^hi^ monocytes only, in female mice [33] suggesting a possible sex difference in response to exercise and antibiotics. In this regard, recent evidence points toward structural and functional sex differences in rodent microglia [66]. In addition, in the study from Möhle and colleagues, the possible region-specific effects may have been diluted, in contrast to our study, where analysis of microglia was restricted to the hippocampus. Moreover, another factor to consider when comparing our results with previously published observations is that we did not use metronidazole in the antibiotic cocktail. Metronidazole can cross the blood brain barrier (BBB) [67] and is used as a model of neurotoxicity in rats [68]. Mass spectrometry analysis allowed us to confirm that none of the antibiotics used in this study reached the hippocampus after 10 weeks of antibiotics treatment (Fig. S5). This supports previous findings [19] suggesting that our observations are mainly a consequence of the disruption of the gut microbiota without a direct effect of antibiotics on the brain per se. However, we cannot rule out potential off-target actions that may contribute to the observed phenotype. We found that exercise failed to reduce the antibiotics-induced neuroinflammatory state. It has been shown that exercise reduced microglia proliferation in aged mice [69] but had no effect in a mouse model of depression [70]. Another study showed that exercise mainly targeted astrocytes in a mouse model of Alzheimer’s disease [71], thus inconsistent reports on the effect of exercise on microglia suggest that further investigation is needed.

To date, the impact of voluntary exercise on the caecal metabolome in a model of gut microbiota disruption remains unexplored, while it has been reported that the main caecal metabolites affected by long term forced exercise in female rats are amino acids [72]. We found enrichment in several amino acid-related pathways among metabolites differentially expressed following antibiotics treatment in sedentary animals but not in exercised male rats. Indeed, the effects of voluntary exercise on the caecal metabolome were few and all but absent in the hippocampal metabolome. Moreover, the dramatic effects of antibiotics treatment on the caecal metabolome are likely to have masked the more subtle effects of voluntary exercise. The only metabolite to be robustly differentially expressed as a function of exercise but also antibiotics treatment was ethyl 2-(4-oxo-4,5-dihydro-1,3-thiazol-2-yl)acetate (PubChem ID: 658099). To date and to the best of the authors’ knowledge, only one publication mentions this compound (by a synonymous name), wherein it is used as an active methylene reagent in the production of azo dyes (colourant), with possible antimicrobial activity [73]. Xenometabolites as a general class have previously been found to be decreased in human body fluids after exercise [74]. It is interesting to note, that its expression correlated with plasma corticosterone (positively), hippocampal BDNF protein (negatively), surviving new-born neurons and newly born neurons in the hippocampus (negatively) as well as with behavioural tasks, including the AHN-dependent MSLR (nominally significant). Our results show that 86% of all quantified metabolites were differentially expressed in the Sed+ABX vs Sed comparison, implicating aminoacyl-tRNA biosynthesis, the citric acid cycle, and several amino acid-related pathways. This is unsurprising given the established link between gut microbiota species and levels of individual circulating metabolites belonging to these pathways and compound classes [75]. Despite the considerable effect of gut microbiota disruption on the caecal metabolome, few metabolites in the hippocampus were altered by antibiotics suggesting that the hippocampus may largely be protected from drastic peripheral changes. Previously, it has been shown that there were 38 differentially expressed metabolites in the cerebrum of germ-free male mice compared to wild-type mice suggesting an interaction between microbiota and brain metabolome [76]. This is in contrast to our focus on the hippocampus only, thus there may be region-specific effects of gut microbiota depletion and exercise or other communication pathways such as blood-brain barrier integrity [77, 78], the vagus nerve [79], SCFA or neuropeptides [1, 80].

An exception was TMAO, a hepatic metabolite produced from gut microbiota catabolism. TMAO is implicated in inflammatory states [81], BBB function [82], age-related cognitive impairment [83] and could also contribute to Alzheimer’s disease pathology by accelerating amyloid aggregation [84] and impairing synaptic plasticity [85]. Indeed, TMAO is present in human cerebrospinal fluid and associated with Alzheimer’s biomarkers and neurodegeneration [86, 87]. However here, the loss of TMAO as a function of antibiotics treatment was not accompanied by cognitive improvements or reduced inflammation, suggesting the relationship is more complex. Moreover, the antioxidant ergothioneine, a TMAO precursor, is known to increase AHN via TrkB signalling [88, 89] and could improve hippocampus-dependent memory in a neurotrophin-dependent manner [90]. Overall, despite antibiotics broadly impacting the gut metabolome, we could not identify any direct association between the gut metabolome and hippocampal metabolome that could explain the behavioural findings. However, it is possible that natural variations in microbiota composition, along with exercise, could have measurable cognitive effects through these metabolites.

The beneficial effect of exercise on cognition has been widely explored in healthy rodents [16] and in models of cognitive decline [91–93]. However, the interaction between exercise and gut microbiota on behaviour remains poorly documented. Previous reports reveal that gut microbiota is involved in hippocampal-dependent behaviours. For example, long term antibiotics exposure induced impairment in the Morris water maze (MWM) in male mice [94] and male rats [28]. To our knowledge we are the first to report that microbiota depletion reduced pattern separation, which is reliant on AHN. FMT approaches have bolstered the notion that gut microbiota are instrumental in hippocampal-dependent cognitive function reliant on AHN [2, 95]. For instance, transfer of a microbiome from Alzheimer’s patients into healthy young male rats impaired their performance in a pattern separation and MWM tasks [2]. The fact that we did not observe an increase in pattern separation by exercise alone is in line with a recent report by the developers of this behavioural task who have highlighted its sensitivity only to interventions that reduce AHN [49]. It is notable however, that exercise rescued the antibiotics-induced impairment in pattern separation, suggesting that the ability of exercise to enhance pattern separation may be a function of gut microbiota integrity.

In relation to anxiety-related behaviours, antibiotics increased the latency to first approach the centre in the NSFT which is reliant on AHN [51, 96]. Similar results were observed in the EPM where a decrease in time spent in the open arms as a result of microbiota depletion was prevented by exercise. These results are in line with previous findings showing that 3 weeks of antibiotics treatment increased anxiety behaviours in the openfield in adult male mice [97]. Exercise alone did not affect anxiety-like behaviours. These results are not surprising as most of the anxiolytic effect of exercise have been described in rat models of pathological states such as post-traumatic stress disorder [98]; restraint stress [99]; or in ageing [100]. Finally, we found that exercise increased the swimming time in the FST in rats treated with antibiotics. Previous study showed that microbiota depletion with antibiotics decreased swimming behaviour in male rats [28] and that a change in microbial diversity is associated with an increased immobility score in FST in male rats [101]. It is interesting to note that the antibiotics cocktail did not induce any body weight change, suggesting that this model did not alter the general health of the animal, similar to previous findings [19, 56].

The relationship between the gut microbiota and AHN has been increasingly explored in recent years [33, 102, 103]. Increased [32] or decreased [104] AHN has been observed in germ-free mice, dependent on sex and age, suggesting a complex interaction between the microbiome and AHN. Interestingly, aberrant AHN coupled with inflammatory changes in the hippocampus was reported in male mice of a mouse model of inflammatory bowel disease [105], a condition in which defective regulation of gut microbiota is evident [106]. Similar to our findings, antibiotics has been shown to decrease AHN in female mice, which was reversed by exercise [33]. The capacity for exercise to enhance AHN is well established [26]. Under baseline conditions, we did not observe a robust effect of exercise on the survival of new neurons (BrdU/NeuN), but the number of immature neurons (DCX) was significantly increased by exercise. Interestingly, we found that the antibiotics-induced reduction of BrdU/NeuN cells was specific to the dorsal hippocampus and that exercise restored this antibiotics-induced decrease in AHN. The dorsal hippocampus plays a role in cognitive behaviours [107] and it has been showed that a neuroinflammatory-induced decrease in neurogenesis in the dorsal hippocampus impaired pattern separation in male rats [108]. We also observed that exercise increased BDNF levels in both plasma and hippocampus irrespective of antibiotics, which could be an important mechanism by which exercise maintains AHN. A previous study has shown that antibiotics decreased mRNA expression of BDNF in mouse hippocampus [109]. We did not observe an effect of antibiotics on BDNF at protein level, which implicates the translation to protein expression as a potential mechanism which negates the effect of microbiota depletion on BDNF. Interestingly, germ-free mice displayed lower protein [110] and mRNA [111] levels of BDNF in the hippocampus. Thus, it is likely that that this reduction in BDNF is due to developmental issues due to the complete lack of microbiome in germ-free mice from birth, in contrast to the changes in the gut microbiome in adulthood that we have induced using antibiotics in the current study. It is important to note that exercise is known to regulate other peripheral factors that cannot be ruled out in the understanding of the gut-brain axis communication as they have an effect of AHN such as circulating hormones (leptin and adiponectin [53]), metabolites (β-hydroxybutyrate [112]) and serum lactate [17].

Our study has certain limitations that must be acknowledged. The pattern separation test (MSLR) employed in our study may not adequately capture the exercise-induced increase in AHN, because measuring improvements in pattern separation in rodents can be challenging [49]. Furthermore, it is crucial for future research to explore the interaction between exercise and the gut microbiota in female rodents considering the sex differences in response to exercise in rats [113, 114] and sex differences in AHN in germ-free mice [104].

Neurogenesis in the adult human hippocampus is still debated [115, 116] and many questions related to its function and regulation remain unanswered [117]. It is not possible to measure the effects of both exercise and a disrupted microbiome on AHN in live human subjects, thus rodent studies are necessary to expand our understanding of the role of gut microbiota in AHN and related behavioural changes. In line with this, a recent study from our laboratory showed that an Alzheimer’s phenotype, including decreased AHN and pattern separation, could be transferred from human Alzheimer’s patients to rats through the gut microbiota [2], confirming that the gut microbiota composition can impact upon cognitive function. Our study contributes to knowledge of the impact of exercise on gut-mediated changes in brain function by showing that it can mitigate cognitive impairment caused by antibiotics-induced gut dysbiosis. Antibiotics are widely prescribed and while their overuse and misuse contribute to the development of antibiotic-resistant bacteria [118], their long-term or repeated usage could also induce cognitive impairment by disrupting the microbiome. In addition, we show that despite the strongly disrupted microbiome, exercise could still exert beneficial effects, suggesting that some of the effects of exercise are independent of the microbiome, and that the use of exercise as a therapy or exercise mimetic [119] may be possible even in the context of disease with disrupted microbiome.

To conclude, our data demonstrate that long-term antibiotics treatment recapitulates the impairments of a disrupted gut microbiota, similar to that of germ-free rodents but without the neurodevelopmental impact of the absence of microbiota during the perinatal period [120]. We observed that exercise partially reversed behavioural and neurogenic changes induced by gut microbiota disruption and that these impairments occurred without significant changes in the hippocampal metabolome, despite drastic shifts in the caecal metabolome. Taken together, these data highlight the importance of the gut microbiota in AHN-dependent behaviours and demonstrate the power of lifestyle factors such as voluntary exercise to attenuate these changes.

### Supplementary information

Supplementary information

Supplementary material legends

Supplementary Figure 1

Supplementary Figure 2

Supplementary Figure 3

Supplementary Figure 4

Supplementary Figure 5

Supplementary table 1

Supplementary table 2

Supplementary table 3

Supplementary table 4

Supplementary table 5

Supplementary table 6

Supplementary table 7

Supplementary table 8

## Data Availability

All code and original data supporting the current study are available upon request.
