# Supplementary material for: Exercise mitigates a gut microbiota-mediated reduction in adult hippocampal neurogenesis and associated behaviours in rats
Source: Transl Psychiatry. 2024 Apr 24;14:195. doi: 10.1038/s41398-024-02904-0 (PMC11043361; doi:10.1038/s41398-024-02904-0)
Supplement: Supplementary file 1 — Supplementary information [file 41398_2024_2904_MOESM1_ESM.docx]

**Exercise mitigates a gut microbiota-mediated reduction in adult hippocampal neurogenesis and associated behaviours in rats**

Sarah Nicolas^1, 2^, Sebastian Dohm-Hansen^1, 2^, Aonghus Lavelle^1, 2^, Thomaz F.S. Bastiaanssen^1,2^, Jane A. English^1,3^, John F. Cryan ^1, 2^, Yvonne M. Nolan^1, 2^ *

1 Department of Anatomy and Neuroscience, University College Cork, Ireland.

2 APC Microbiome Ireland, University College Cork, Ireland.

3 INFANT Research Centre, Cork University Hospital, Wilton, Cork, Ireland.

Corresponding author * Y. M. Nolan

Department of Anatomy and Neuroscience

University College Cork, Ireland

Tel: +353 21 420 5476

Email: [y.nolan@ucc.ie](mailto:y.nolan@ucc.ie)

**Email:**  y.nolan@ucc.ie

Supplementary Information

**Gut microbiota disruption increased microglial activation.**

Two-way ANOVA revealed that exercise [F_(1, 16)_ = 5.231, p=0.0361] and antibiotics [F_(1, 16)_ = 48.17, p<0.0001] had an overall effect on the microglia soma size but that the interaction between both factors was not significant [F_(1, 16)_ = 0.02156, p=0.8851]. We observed that antibiotics increased the soma size in sedentary rats (Sed vs Sed+ABX p=0.0007) and to a lesser extent in exercised rats (Ex vs Ex+ABX p=0.001; **Fig S4a,f**). Next, we classified the microglia soma size as previously described (1). Two-way ANOVA showed that both exercise [F_(1, 16)_ = 6.023, p=0.0260] and antibiotics [F_(1, 16)_ = 39.82, p<0.0001] affected the percentage of microglia with a small soma size but there was no interaction [F_(1, 16)_ = 0.1229, p=0.7305] (**Fig S4b**). We observed a similar result for the percentage of microglia with a large soma size, where exercise [F_(1, 16)_ = 4.848, p=0.0427] and antibiotics [F_(1, 16)_ = 40.24, p<0.0001] significantly affected the percentage of microglia having a large soma but without an interaction [F_(1, 16)_ = 0.0200, p=0.8893] (**Fig S4c**). The increased cell soma size induced by antibiotics was concomitant with an overall effect of antibiotics on the number of microglia in the whole hippocampus [F_(1, 16)_ = 9.441, p=0.0073]. Neither exercise [F_(1, 16)_ = 0.5648, p=0.4632] or the interaction between exercise and antibiotics [F_(1, 16)_ = 0.6843, p=0.4203] affected the number of microglia per mm^2^ in the hippocampus (**Fig S4d**). Finally, we used a distribution analysis approach based on the classification of the microglia into range of soma size (2) to reveal a right-shifted distribution for soma size in microglia from antibiotic-treated rats confirming their increased soma size (**Fig S4e**). Interestingly, in the exercise antibiotics group we observed that the distribution shift slightly towards the left, suggesting a subtle effect of exercise on reducing the activation state of the microglia by antibiotics.

**Supplementary methods**

**Antibiotic administration**

To deplete the gut microbiota, a combination of antibiotics was chosen based on a previous report that this combination reduced the faecal bacterial DNA load by 400-fold while ensuring the animals’ health (3), and that vancomycin and imipenem are minimally absorbed through oral administration (4,5) while ampicillin is only absorbed in fasting conditions (6). Antibiotics were administered in the drinking water for the duration of the experiment and bottles were changed every second day. The antibiotic cocktail consisted of ampicillin (1 g/L; #69-52-3; Discovery Fine Chemicals Ltd), vancomycin (0.5 g/L; #1404-93-9; Discovery Fine Chemicals Ltd) and imipenem (0.250 g/L; #74431-23-5; Discovery Fine Chemicals Ltd). Water intake and body weight were monitored daily for the first 2 weeks to ensure the health of the animals, and subsequently monitored twice a week until the termination of the study.

**Behaviours**

*Modified spontaneous location recognition test*

The task was conducted in a circular arena (90 cm diameter) under dim light conditions (30 lux), with the floor covered with standard bedding. The arena was surrounded by three proximal spatial cues and distal standard furniture. Rats were habituated to the circular arena for 10 min per day for 5 consecutive days before testing. During the acquisition phase on day 6, rats were placed in the arena with three identical objects placed 15 cm from the wall and 30 cm from the centre of the arena. For the large separation task, three identical objects (1, 2, and 3) were separated by 120° and 49 cm apart (Fig. 2a), and for the small separation task (pattern separation) two of the objects were separated by 50 ° and 20.5 cm apart (2, 3), and the third object at an equal distance between the two (1; Fig. 2b). Rats were left to explore the arena with the objects for 10 min and 24 hours later the test phase took place. During the test, two objects (identical to the objects used during the acquisition phase) were positioned such that one of the objects was in familiar location (1), while one was in a novel location (4). The novel location was chosen such that the object was positioned exactly in between the two familiar locations of the acquisition phase. Two sets of objects were used; one for the large separation task and one for the small separation task. Rats were allowed to explore for 5 min. Both object sets as well as the ordering and locations were counterbalanced within and between groups. Scores are presented as discrimination index, which is DI=$\frac{time \left( novel \right)-time(familiar)}{time(novel+familiar)}$ (7). 9 rats per group were tested on this task.

*Y-maze*

The maze consisted of three arms 120 ° from each other (40 × 10 × 20 cm) (Fig 2c). The testing room had three proximal spatial cues. The rat was placed in the distal end of one of the open arms (start/familiar arm), facing away from the centre of the maze. The animal was allowed to explore the start arm and a second arm for 5 minutes, while access to the third arm was blocked, and then returned to its home cage for 2 hours. The rat was then placed back in the start arm and allowed to explore all 3 arms of the maze for 5 min. The total time spent in each arm was recorded for the first 2.5 min and a discrimination ratio (DR) of novel arm investigation was calculated as DR = $\frac{time\left( novel \right)}{time(novel+familiar)}$. In order to test animals within the 2 hours interval time, only 9 rats per group were tested in this task. Two rats (one in the exercise group, and one in the exercise+antibiotics group) were removed from the analysis because they escaped from the maze more than two times during the 2^nd^ trial. Scoring was analysed during the first 2.5 minutes of the task to capture the investigative behaviour of all of the rats, It has been shown that the investigation time of a novel location (or object) is highest the first 3 minutes of a test (8–10).

*Novelty suppressed feeding*

The testing apparatus consisted of an arena (90 cm diameter) with 2 cm of wooden bedding on the floor and a single food pellet placed on a white plastic support in the centre, under a bright light (1000 lux) environment (Fig 2d). Rats were fasting overnight prior to testing and then placed close to the arena wall. The latency to first approach the centre and the latency to eat (defined as the rat biting the pellet) was recorded. Between each rat exposure to the arena, the bedding was cleaned (fecal pellets removed); ¼ of it was changed and then thoroughly mixed to remove possible odor cues. Immediately after the test, food consumption was measured for 30 min (+/- 1min) to control for potential feeding differences. The food consumption was not measured when it was outside of the 30min time window.

*Elevated Plus Maze*

The maze consisted of two open arms (51 × 10 cm) and two enclosed arms (51 × 10 × 41 cm) extending from a common central platform (10 × 10 cm) that was elevated 55 cm above the floor on a central pedestal (Fig 2e). The open arms of the maze are considered to be more aversive for rodents than the closed ones, and anxiolytic behaviour is defined as increased number of entries and time spent in the open arms (11). Each rat was placed in the centre of the maze facing an open arm and left to explore for 5 min under red light condition (5 lux). An arm entry was defined as the four paws of the animal being within the borders of the arm. The percentage time and entries in the open arms (open/(open + closed) was calculated from the raw data, as an index of anxiety. One rat from the control group and two rats from the exercise group jumped/fell from the maze, and one rat from the antibiotics group did not exit the closed arm; data from these rats were removed from the analysis.

*Forced Swim Test*

A pre-swim (15 min) was conducted 24 hours prior to the test swim. On the test day, all animals were introduced again to the glass cylinder (46 cm tall × 21 cm in diameter) filled with water (24 °C) to a depth of 30 cm (Fig 2f). Test sessions (5 min) were recorded by a video camera positioned directly above the cylinder. For analysis, a time-sampling technique was employed, whereby the predominant behaviour (immobility, swimming, or climbing) in each 5-second period of the 5-minute interval was recorded as previously described (12).

**Post-mortem analysis**

**Immunohistochemistry**

*Adult hippocampal neurogenesis – BrdU/NeuN staining*

To assess newborn hippocampal neuron survival and maturation, free-floating sections (40 μm) from the dentate gyrus (DG) were immunohistochemically stained for BrdU/NeuN (survival of new neurons). Sections were incubated in HCl (2 M) for 45 min at 37 °C and renatured in 0.1 M sodium tetraborate. All sections were blocked in 10% normal donkey serum (#S30-M ; Sigma) and incubated with rat anti-BrdU (1:1000; #ab6326; Abcam) and mouse anti-NeuN (1:2000; #MAB377; Millipore) overnight at 4 degrees. Sections were incubated in the appropriate AlexaFluor secondary antibody (AF488 or AF564) and then with Dapi (0.1µg/ml; # D9542; Sigma) to stain nuclei. Lastly, sections were washed, mounted, and coverslipped with anti-fade mounting media (#HC08; Sigma).

*Newly born neurons - DCX staining*

To analysis neuroblast number and morphology, DG sections were stained for doublecortin (DCX), a maker for young immature neurons. All sections were blocked in 10% normal donkey serum and incubated with rabbit anti-DCX (1:5000; #ab18723; Abcam) 48 hours at 4 degrees. Sections were incubated in the appropriate AlexaFluor secondary antibody (donkey anti-rabbit AF488) and then with Dapi to stain nuclei. Lastly, sections were washed, mounted, and coverslipped with anti-fade mounting media.

*Microglia – Iba1*

To analysis microglia number and size, DG sections were stained for ionized calcium-binding adapter molecule 1 (Iba1), a microglia/macrophage-specific calcium binding protein(1). All sections were blocked with 10% normal donkey serum and then incubated with rabbit Iba1 primary antibody (1:500; #190-19741; Wako) overnight at 4 degrees. Sections were incubated in the appropriate AlexaFluor secondary antibody (donkey anti-rabbit AF488) and then with Dapi to stain nuclei. Lastly, sections were washed, mounted, and coverslipped with anti-fade mounting media.

*Imaging, cell quantification and cell morphology analysis*

Images (with blinded identifiers) were obtained using an Olympus AX70 upright microscope (10X and 20X objective) (BioSciences Imaging Centre, Department of Anatomy and Neuroscience, UCC).  BrdU+/NeuN+ and DCX+ cells were counted on both hemispheres of each section, from 5 rats per group. The dorsal hippocampus was defined as AP: Bregma − 2.2 to − 5.2 and ventral hippocampus as AP: − 5.2 to − 6.7. On average 10 sections were analysed per brain (approximately 75% dorsal and 25% ventral sections). Data are expressed as cells per mm^2^. To assess the degree of microglial activation the number of Iba-1+ cells per section and the soma size of Iba-1^+^ cells in the DG were analyzed(13). Six sections from 5 rats per group were analysed using Image J and the number of cells per section was expressed as cells/mm^2^. For the cell soma size, twenty randomly selected cells were sampled per section and 6 sections were analyzed per animal in 5 rats per group. The area of the soma was measured using ImageJ and expressed as μm^2^. For microglial classification, Iba1+ cells with soma area equal to or below one standard deviation above the mean of the control group were categorized as ‘small soma size’ and cells with a soma area above one standard deviation above the mean of the control group were categorized as ‘large soma size’(1).

**ELISAs**

*Corticosterone assay*

Plasma corticosterone levels were assayed in duplicate using a corticosterone immunoassay kit according to the manufacturers’ instructions (#ADI-900-097; Enzo Life Sciences, UK).

*Cytokines*

The concentration of cytokines (interleukin-1β (IL-1β), IL-4, IL-6, IL-10, tumour necrosis factor-α (TNF-α) and interferon-γ (IFNγ)) was measured in plasma taken at the end of the study using an electrochemoluminescence (ECL)-based assay (#K15059D, MesoScale Discovery, USA) following the manufacturer’s instructions. Only IL-6 and TNF-α were above the detection threshold.

*BDNF*

The concentration of BDNF was measured in plasma and hippocampal lysate by ELISA following the manufacturer’s instructions (#ERBDNF; Thermo Scientific). Total protein was extracted from hippocampal tissue using lysis buffer (50 mM Tris-HCl, pH 7.5, 150 mM NaCl, 5 mM EDTA containing 0.5% Triton X-100) in combination with Complete^TM^ Mini EDTA-free protease inhibitors (Roche) in a 1:1 ratio with dH_2_O. Total protein in each sample was determined by the Bradford method (BioRad). BDNF was normalized to the total protein concentration of each sample.

With regard to analysis of all ELISA data, samples that were not within the range of the respective standard curves were removed from analysis.

**Quantitative Reverse-Transcription PCR (RT-qPCR)**

Muscle and colon tissue was dissected from all animals that were euthanised by decapitation while only samples from two rats per group from those that were transcardially perfused were processed as the others were possibly contaminated with PFA. Total RNA was extracted from frozen muscle and colon tissue using QIAzol lysis reagent (#79306; Qiagen) and treated using a Turbo DNA-free kit (#AM1907; Ambion/life Technologies) as per the manufacturer’s instructions. Total RNA yield and purity were determined using the Nanodrop System (Thermo Scientific). Synthesis of cDNA was performed using the high capacity cDNA reverse transcription kit (#10400745; Applied Biosystems) and SureCycler® 8800 (Agilent Technologies) diluted to a final concentration of 10 ng/μl. All qPCR was performed in 2 technical replicates for each biological sample on a LightCycler® 480 Instrument II (Roche). Primers and associated sequences used are detailed in Supplementary table 1. Each reaction consisted of 1 μl of sample (5 ng/μl), 5 μl of Sybr MasterMix (KiCqStart® SYBR® Green qPCR ReadyMix™ with ROX™ for ABI instruments, # KCQS02; Sigma-Aldrich), 0.1 μl of both forward and reverse primers (detailed in Supplementary table 1), and 3.8 μl of RNase free H_2_O. Tissue samples that failed to show a standard amplification curve were removed from analysis. Relative gene expression was adjusted to β-Actin and Hprt and quantified using the 2^−ΔΔCT^ method.

**Metabolomics analysis**

**Caecal content sample preparation:**

The samples were initially added 4 x wt/vol MQW (MilliQ-water) and subsequently vortexed and centrifuged for 10 min at 16.000 g and 4 °C. Next the supernatant was collected, filtered by centrifugation (5 min at 15.000 g 4°C) using spinX filters and finally diluted five times in eluent A1 (10 mM ammonium formate, 0.1% formic acid in water).

**Hippocampal sample preparation:**

300 µL precooled MeOH:MQW (1:2 v/v) was added to the samples along with stainless steel beads. Samples were then placed in the precooled blocks and homogenized for 3 x 30 s (blocks were cooled at -20 °C for 2 min between each run) by beat beating, followed by further disruption in an ultrasonic bath for 10 min. Samples were centrifuged for 10 min at 12.000 g and 4 °C. 300 µL of the supernatant were collected in a new eppendorf tube and the residue were reextracted with 200 µL MeOH:MQW (1:2 v/v). The two supernatants were combined and 200 µL chloroform:methanol (1:3, v:v) was added and the samples vortexed.  350 µL of the top (polar) layer was transferred to new Eppendorf tubes and dried under a light flow of N2. The samples were resuspended in 150 µL resuspension mix (Eluent A1 ((10 mM ammonium formate, 0.1 % formic acid in water)) and eluent B1 (10 mM ammonium formate, 0.1 % formic acid in methanol) mixed) mixed 9:1, v/v) and transferred to a labelled spin-filter tube and filtered at 3000 g for 5 min.  Finally, the samples were diluted five times using eluent A1 (10 mM ammonium formate, 0.1 % formic acid in water). All samples were analyzed in a randomized order. The analysis was performed using the same GC device. In both cases, the system was controlled by ChemStation (Agilent). One of the tubes containing sample from the exercise+antibiotics group burst during the sample preparation and was thus removed from the analysis.

**Mass spectrometry and feature identification:**

Sample analysis was carried out by MS-Omics as follows. The analysis was carried out using a Thermo Scientific Vanquish LC coupled to Thermo Q Exactive HF MS. An electrospray ionization interface was used as ionization source. Analysis was performed in negative and positive ionization mode. The UPLC was performed using a slightly modified version of a protocol previously described (14). Peak areas were extracted using Compound Discoverer 3.1 (Thermo Scientific). Identification of compounds were performed at four levels; Level 1: identification by retention times (compared against in-house authentic standards), accurate mass (with an accepted deviation of 3ppm), and MS/MS spectra, Level 2a: identification by retention times (compared against in-house authentic standards), accurate mass (with an accepted deviation of 3ppm). Level 2b: identification by accurate mass (with an accepted deviation of 3ppm), and MS/MS spectra, Level 3: identification by accurate mass alone (with an accepted deviation of 3ppm).

**Bioinformatic analysis:**

Metabolomic analysis was constrained to non-drug-related features annotated at levels 1, 2a, and 2b (n = 652 features in the caecum; n = 215 features in the hippocampus), as provided by MS-Omics and carried out in R (version 4.1.1). All values below the limit of detection (LOD) were set to “*NA*” (i.e. considered missing). Metabolites with greater than 50% missingness across all samples and conditions were removed from the analysis (n = 610 features remaining in the caecum; n = 210 features remaining in the hippocampus). Due to the uneven distribution of missing values between experimental groups (attributed to the antibiotics treatment, particularly in the caecal metabolome), this filtering strategy retained some features with only 1 valid value in a given condition for quantitation. However, this simultaneously allowed us to identify differentially regulated features with few above-threshold values (**Fig 5c**). Principal component analysis (PCA) was used to visualize caecal and hippocampal metabolomics, using the *ade4* and *factoextra* packages. Metabolite feature peak areas were normalized by variance stabilizing normalization (VSN), which was originally developed for microarray data (15) but has subsequently been used for untargeted metabolomics, based on similar data distributions (16) and underlying error model assumptions(17). VSN uses a generalized logarithmic transformation (glog, base 2) to ensure constant variance across abundance (peak area) levels. The glog2 values approach log2 for peak intensities >>0. VSN was applied using the *vsn* package. As VSN assumes that most features are not differentially expressed to perform the normalization, the “lts.quantile” parameter was set to 0.5 to account for the high level of differential expression in the caecum (i.e. allowing for up to 50% outlier features), while the default 0.9 was used for the hippocampal data. Differential expression analysis was performed with *limma*(18), with trend = TRUE and robust = TRUE in the *eBayes* function. Metabolite features with a Benjamini-Hochberg-adjusted p-value (i.e. false discovery rate, FDR) of p < 0.05 were considered significant. Pathway enrichment analysis with differentially regulated caecal metabolites at annotation levels 1 and 2a (the most confident and well-annotated levels) was performed in Metaboanalyst(19), using *Rattus norvegicus* KEGG pathways and a hypergeometric test.

**Statistical analysis**

We performed a priori comparison for the statistical analysis. *A priori*, we expected exercise to affect PGC1⍺ and BDNF (20); antibiotics-induced gut microbiota dysbiosis to affect cytokines level (21); gut physiology (22) and microglia (23); either antibiotics and/or exercise alone to affect corticosterone (24,25) and neurogenesis (26,27). Any *a priori* comparisons were subjected to a Bonferroni correction. Each *n* number represent one rat, *n* number are described in the figure legend for each graph. Sample size was determined by using the software G*Power. The reported *p*-values are adjusted for multiple comparison.

References

1. Kozareva DA, Hueston CM, Ó’Léime CS, Crotty S, Dockery P, Cryan JF, et al. Absence of the neurogenesis-dependent nuclear receptor TLX induces inflammation in the hippocampus. J Neuroimmunol [Internet]. 2019;331(August 2017):87–96. Available from: https://doi.org/10.1016/j.jneuroim.2017.08.008

2. Davis BM, Salinas-Navarro M, Cordeiro MF, Moons L, De Groef L, Groef L De. Characterizing microglia activation: A spatial statistics approach to maximize information extraction. Sci Rep. 2017 May;7(1):1576.

3. Reikvam DH, Erofeev A, Sandvik A, Grcic V, Jahnsen FL, Gaustad P, et al. Depletion of Murine Intestinal Microbiota: Effects on Gut Mucosa and Epithelial Gene Expression. PLoS One [Internet]. 2011 Mar 21;6(3):e17996. Available from: https://doi.org/10.1371/journal.pone.0017996

4. Rao S, Kupfer Y, Pagala M, Chapnick E, Tessler S. Systemic absorption of oral vancomycin in patients with Clostridium difficile infection. Scand J Infect Dis. 2011;43(5):386–8.

5. Bush K. CHAPTER 15 - Other β-lactam antibiotics. In: Finch RG, Greenwood D, Norrby SR, Whitley RJBT-A and C (Ninth E, editors. London: W.B. Saunders; 2010. p. 226–44. Available from: https://www.sciencedirect.com/science/article/pii/B9780702040641000154

6. Kester M, Karpa KD, Vrana KE. 4 - Treatment of Infectious Diseases. In: Kester M, Karpa KD, Vrana KEBT-EIRP (Second E (Second E, editors. Philadelphia: W.B. Saunders; 2012. p. 41–78. Available from: https://www.sciencedirect.com/science/article/pii/B9780323074452000045

7. Reichelt AC, Kramar CP, Ghosh-Swaby OR, Sheppard PAS, Kent BA, Bekinschtein P, et al. The spontaneous location recognition task for assessing spatial pattern separation and memory across a delay in rats and mice. Nat Protoc. 2021;16(12):5616–33.

8. Dellu F, Mayo W, Cherkaoui J, Le Moal M, Simon H. A two-trial memory task with automated recording: study in young and aged rats. Brain Res. 1992;588(1):132–9.

9. Toyoshima M, Yamada K, Sugita M, Ichitani Y. Social enrichment improves social recognition memory in male rats. Anim Cogn [Internet]. 2018;21(3):345–51. Available from: https://doi.org/10.1007/s10071-018-1171-5

10. Dellu F, Contarino A, Simon H, Koob GF, Gold LH. Genetic differences in response to novelty and spatial memory using a two-trial recognition task in mice. Neurobiol Learn Mem. 2000;73(1):31–48.

11. Cryan JF, Sweeney FF. The age of anxiety: role of animal models of anxiolytic action in drug discovery. Br J Pharmacol. 2011 Oct;164(4):1129–61.

12. Slattery DA, Cryan JF. Using the rat forced swim test to assess antidepressant-like activity in rodents. Nat Protoc. 2012;

13. Nicolas S, McGovern AJ, Hueston CM, O’Mahony SM, Cryan JF, O’Leary OF, et al. Prior maternal separation stress alters the dendritic complexity of new hippocampal neurons and neuroinflammation in response to an inflammatory stressor in juvenile female rats. Brain Behav Immun. 2022 Jan;99:327–38.

14. Doneanu CE, Chen W, Mazzeo JR, Ds OR. UPLC/MS Monitoring of Water-Soluble Vitamin Bs in Cell Culture Media in Minutes. Water Appl note. 2011;1–7.

15. Huber W, Von Heydebreck A, Sültmann H, Poustka A, Vingron M. Variance stabilization applied to microarray data calibration and to the quantification of differential expression. Bioinformatics. 2002;18(SUPPL. 1).

16. Li B, Tang J, Yang Q, Cui X, Li S, Chen S, et al. Performance evaluation and online realization of data-driven normalization methods used in LC/MS based untargeted metabolomics analysis. Sci Rep. 2016;6(December):1–13.

17. Rocke DM, Durbin B. A model for measurement error for gene expression arrays. J Comput Biol. 2001;8(6):557–69.

18. Ritchie ME, Phipson B, Wu D, Hu Y, Law CW, Shi W, et al. Limma powers differential expression analyses for RNA-sequencing and microarray studies. Nucleic Acids Res. 2015;43(7):e47.

19. Xia J, Psychogios N, Young N, Wishart DS. MetaboAnalyst: A web server for metabolomic data analysis and interpretation. Nucleic Acids Res. 2009;37(SUPPL. 2):652–60.

20. Wrann CD, White JP, Salogiannnis J, Laznik-Bogoslavski D, Wu J, Ma D, et al. Exercise induces hippocampal BDNF through a PGC-1α/FNDC5 pathway. Cell Metab. 2013;

21. Jang SE, Lim SM, Jeong JJ, Jang HM, Lee HJ, Han MJ, et al. Gastrointestinal inflammation by gut microbiota disturbance induces memory impairment in mice. Mucosal Immunol. 2018;

22. Zarrinpar A, Chaix A, Xu ZZ, Chang MW, Marotz CA, Saghatelian A, et al. Antibiotic-induced microbiome depletion alters metabolic homeostasis by affecting gut signaling and colonic metabolism. Nat Commun [Internet]. 2018;9(1). Available from: http://dx.doi.org/10.1038/s41467-018-05336-9

23. Boehme M, van de Wouw M, Bastiaanssen TFS, Olavarría-Ramírez L, Lyons K, Fouhy F, et al. Mid-life microbiota crises: middle age is associated with pervasive neuroimmune alterations that are reversed by targeting the gut microbiome. Mol Psychiatry. 2020 Oct;25(10):2567–83.

24. Lapmanee S, Charoenphandhu J, Teerapornpuntakit J, Krishnamra N, Charoenphandhu N. Agomelatine, venlafaxine, and running exercise effectively prevent anxiety- and depression-like behaviors and memory impairment in restraint stressed rats. PLoS One. 2017;12(11):1–23.

25. Desbonnet L, Clarke G, Traplin A, O’Sullivan O, Crispie F, Moloney RD, et al. Gut microbiota depletion from early adolescence in mice: Implications for brain and behaviour. Brain Behav Immun [Internet]. 2015;48:165–73. Available from: internal-pdf://166.67.96.228/Gut microblota behavior BBI2015.pdf

26. Möhle L, Mattei D, Heimesaat MM, Bereswill S, Fischer A, Alutis M, et al. Ly6Chi Monocytes Provide a Link between Antibiotic-Induced Changes in Gut Microbiota and Adult Hippocampal Neurogenesis. Cell Rep [Internet]. 2016 May;15(9):1945–56. Available from: internal-pdf://199.212.82.23/1-s2.0-S2211124716305186-main.pdf

27. O’Leary JD, Hoban AE, Murphy A, O’Leary OF, Cryan JF, Nolan YM, et al. Differential effects of adolescent and adult-initiated voluntary exercise on context and cued fear conditioning. Neuropharmacology. 2019;
