# Supplementary material for: Exercise mitigates a gut microbiota-mediated reduction in adult hippocampal neurogenesis and associated behaviours in rats
Source: Transl Psychiatry. 2024 Apr 24;14:195. doi: 10.1038/s41398-024-02904-0 (PMC11043361; doi:10.1038/s41398-024-02904-0)
Supplement: Supplementary file 2 — Supplementary material legends [file 41398_2024_2904_MOESM2_ESM.docx]

**Supplementary Figure Legends**

**Supplementary figure 1: Metabolomic analysis of caecal content**

**a-** Caecum weight adjusted to rat body weight (n=10; two-way ANOVA; main effect of Abx: *a priori* comparisons $$$p<0.001). **b-**  Representative picture of caecum **c-** Volcano plot of caecal metabolites in Ex+ABX and Sed+ABX rats. **d-** Normalised peak areas for selected caecal content metabolites (all comparisons, *p* > 0.05) ***p<0.001 (FDR-adjusted; Limma *p*-value) **Abbreviation:** FC – fold change; glog_2_ – generalised logarithm base 2.

**Supplementary figure 2: Metabolomic analysis of hippocampus**

**a-** Principal Component Analysis (PCA) of hippocampal metabolomics. **b-** Volcano plot of hippocampal metabolites for Sed and Sed+ABX rats (labelled metabolites have *p*_FDR-adjusted_ <0.05). **c-** Volcano plot of hippocampal metabolites in Sed and Ex rats. **d-** Volcano plot of caecal metabolites in Ex+ABX and Sed+ABX rats. **e-** Normalised peak areas for the metabolite Trimethylamine N-Oxide (TMAO) in the hippocampus. Sed vs ABX rats (glog2 fold change = -4.9; *p*_FDR-adjusted_ = 1.5E-5) **f-** Normalised peak areas for the metabolite Ethyl 2-(4-oxo-4,5-dihydro-1,3-thiazol-2-yl)acetate in the hippocampus (all comparisons, *p* > 0.05). **g-** Normalised peak areas for selected hippocampal metabolites. ***p<0.001 (FDR-adjusted; Limma *p*-value) - **Abbreviation:** FC – fold change; glog_2_ – generalised logarithm base 2.

**Supplementary figure 3: Behavioural parameters**

**a-** Distance travelled (m) in the 2 trials of the Y-maze task (n=8-9). **b-** Body weight loss normalised to individual body weight after overnight fasting before the novelty suppressed feeding task (n=10). **c-** Pearson correlation for latency to eat versus % body weight loss for rats in the sedentary group (r_pearson_ = 0.59, *p* = 0.072), exercise group (r_pearson_ = -0.7803, *p* = 0.008), antibiotics group (r_pearson_ = -0.333, *p* = 0.347) and exercise+antibiotics group (r_pearson_ = -0.3681, *p* = 0.295). **d-** Pearson correlation for latency to approach the centre versus body weight loss for rats in the sedentary group (r_pearson_ = -0.2314, *p* = 0.520), exercise group (r_pearson_ = -0.6096, *p* = 0.061), antibiotics group (r_pearson_ = 0.4372, *p* = 0.206) and exercise+antibiotics group (r_pearson_ = 0.2281, *p* = 0.526).

**Supplementary figure 4: Analysis of microglia activation state**

**a-** Microglia soma size (μm^2^) in the hippocampus (n=5; two-way ANOVA; main effect of abx; $$p<0.01; $$$p<0.001; main effect of Ex). Classification of microglia for **b-** small soma size (n=5; two-way ANOVA; main effect of Abx $$p<0.01; main effect of Ex) and **c-** large soma size (n=5; two-way ANOVA; main effect of abx; $$p<0.01; main effect of Ex). **d-** Number of Iba1 positive cells per mm^2^ in the whole hippocampus (n=5; Two-Way ANOVA; Main effect of Abx). **e-** distribution analysis of soma area in the hippocampus. n=5 **f-** Representative images of microglia. Data are graphed as means + SEM.

**Supplementary figure 5: Antibiotics content of caecum and hippocampus**

Detection peak area (arbitrary unit) for ampicillin and vancomycin by MS/LS. Imipenem was not detectable in any of the samples. n=10 for caecum samples and n=4-5 for hippocampal samples.

**Supplementary table 1: Primers used for RT-qPCR**

Abbreviations: IL – Interleukin ; TNF – Tumor Necrosis Factor ; CCL – Chemokine Ligand ; PGC1α - Peroxisome proliferator-activated receptor gamma coactivator 1-alpha; Hprt - hypoxanthine phosphoribosyl transferase 1

**Supplementary table 2: Differentially regulated Trimethylamine-N-oxide (TMAO) precursors -** Measured in the hippocampus and caecum in Sed + Abx vs. Sed-rats. NB: (-) Undetected, FC – Fold change

**Supplementary table 3: Pathway analysis of caecal metabolites (annotation levels 1 & 2a) differentially regulated by antibiotics** (Antibiotics vs. Sedentary; FDR < 0.05; ↑Up-regulated; ↓down-regulated)
