# Supplementary figures and images for: Exercise mitigates a gut microbiota-mediated reduction in adult hippocampal neurogenesis and associated behaviours in rats
Source: Transl Psychiatry. 2024 Apr 24;14:195. doi: 10.1038/s41398-024-02904-0 (PMC11043361; doi:10.1038/s41398-024-02904-0)

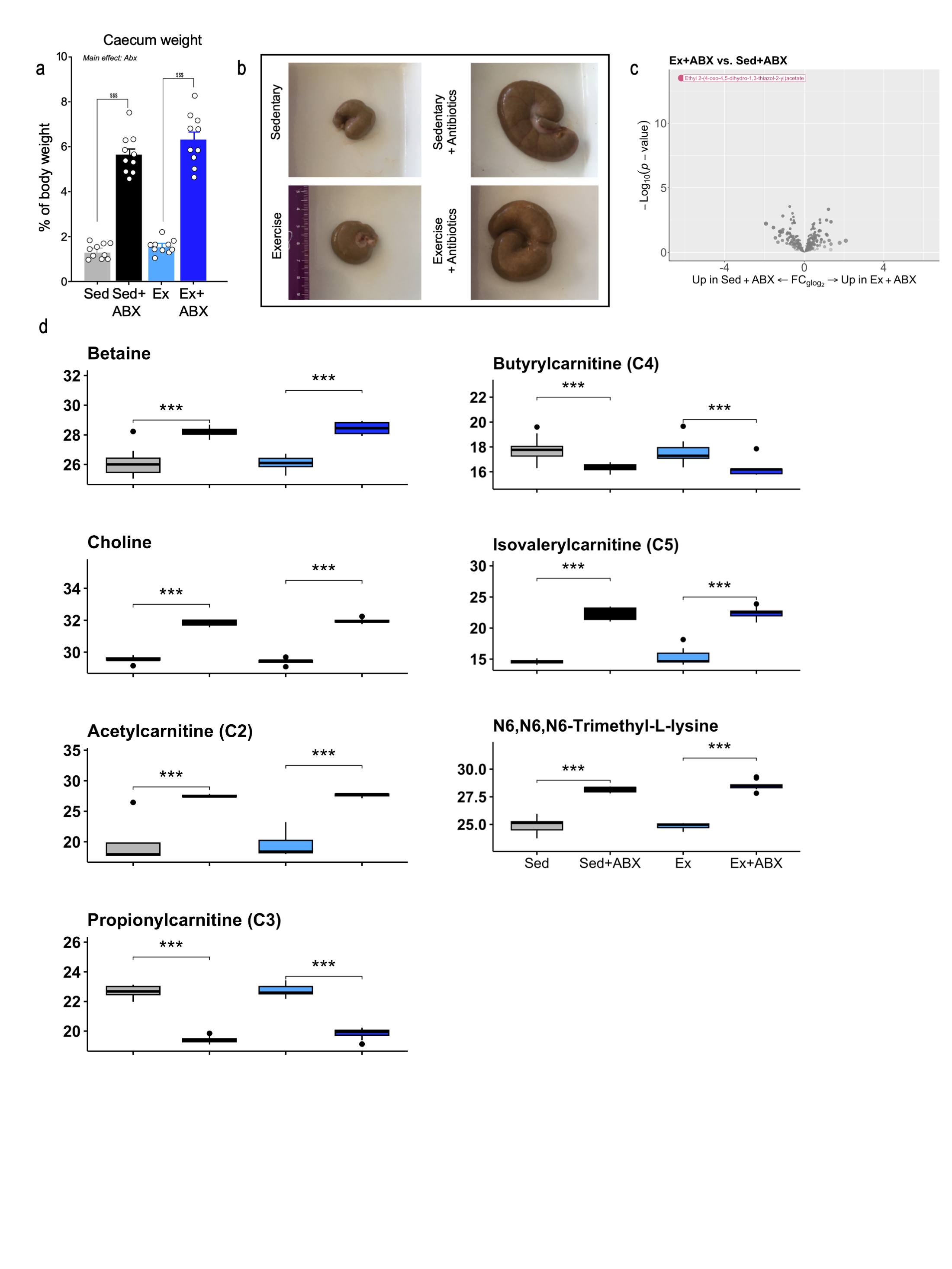

Supplement: Supplementary file 3 — Supplementary Figure 1 [file 41398_2024_2904_MOESM3_ESM.tif]

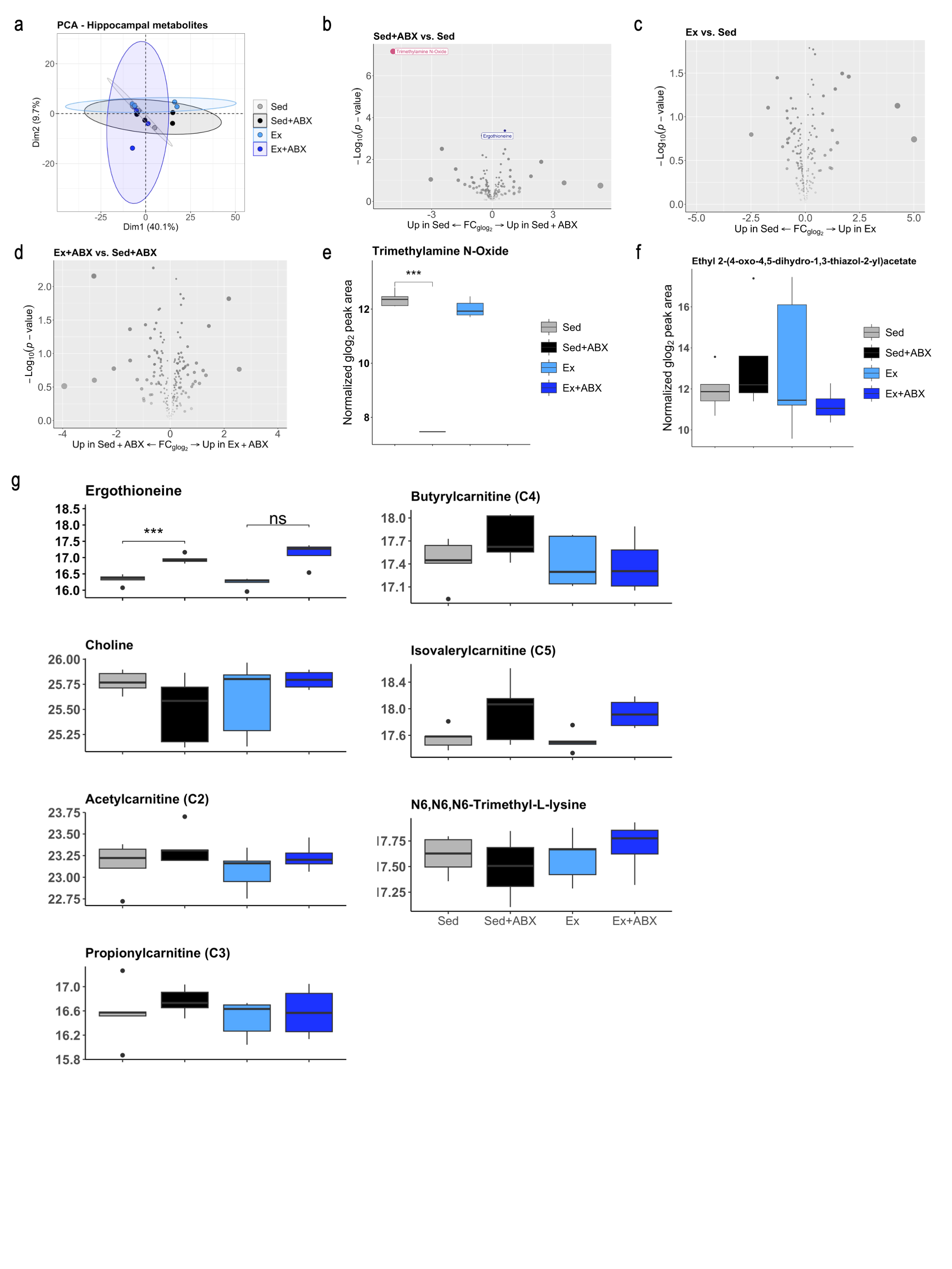

Supplement: Supplementary file 4 — Supplementary Figure 2 [file 41398_2024_2904_MOESM4_ESM.tif]

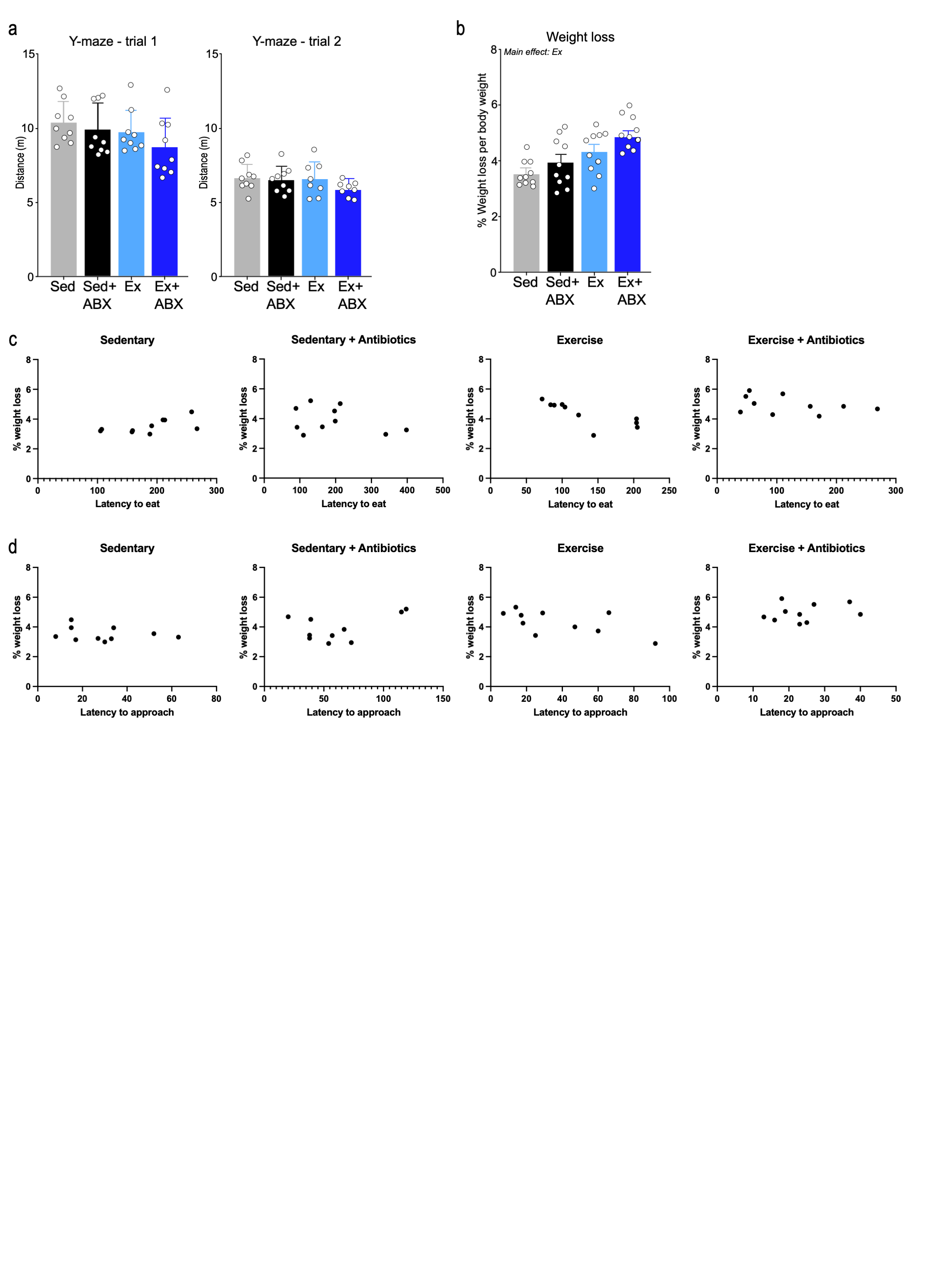

Supplement: Supplementary file 5 — Supplementary Figure 3 [file 41398_2024_2904_MOESM5_ESM.tif]

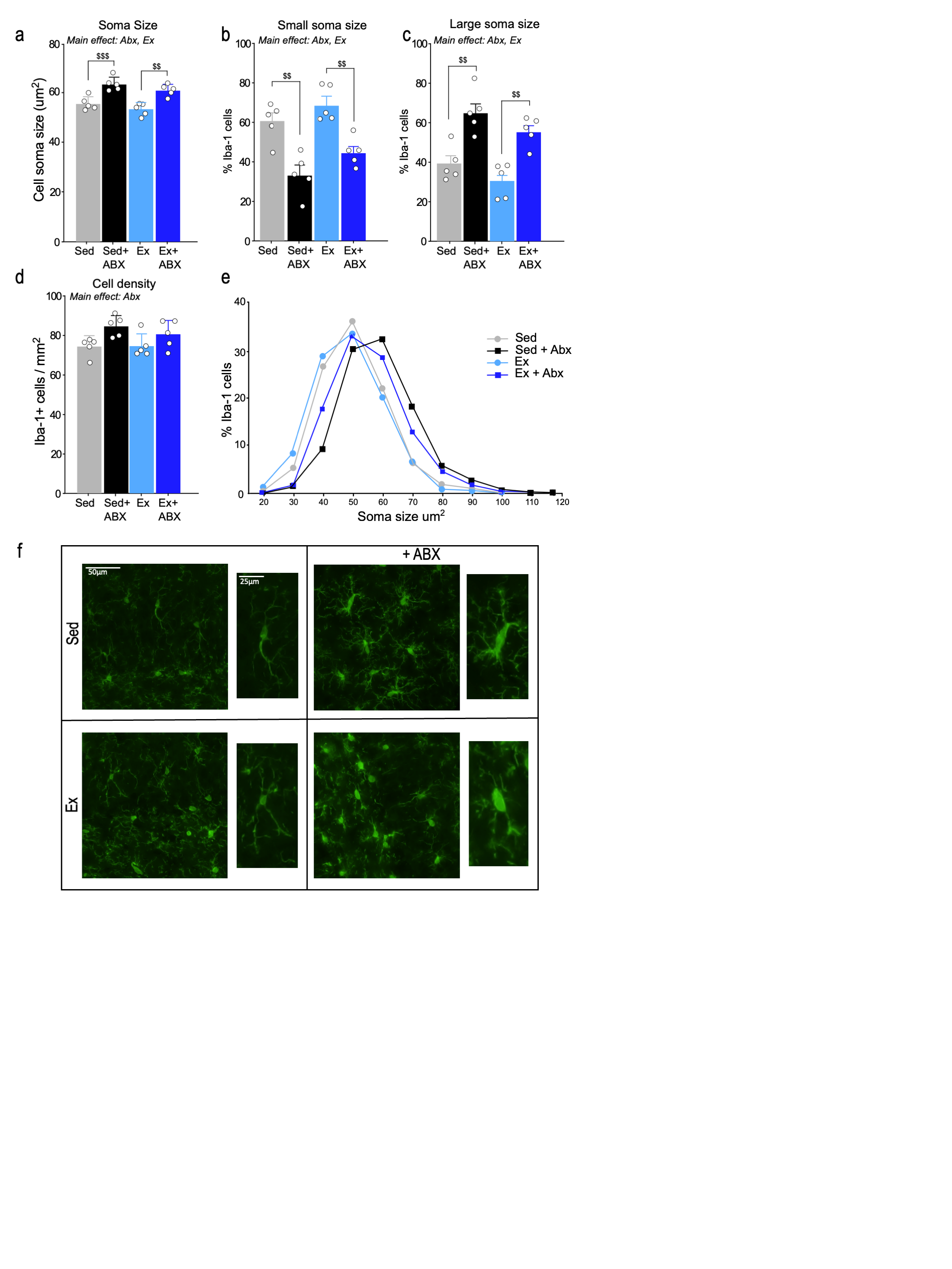

Supplement: Supplementary file 6 — Supplementary Figure 4 [file 41398_2024_2904_MOESM6_ESM.tif]

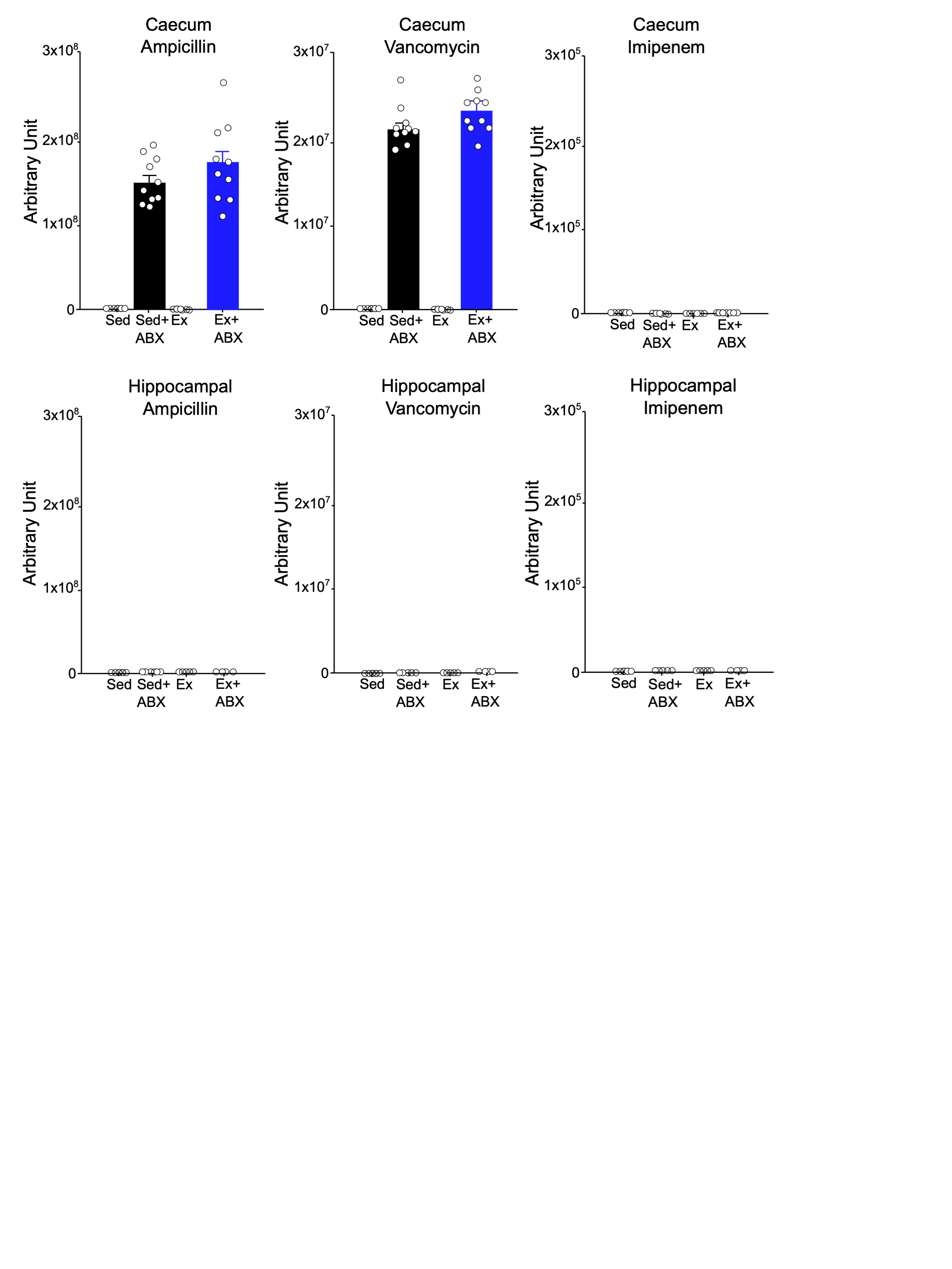

Supplement: Supplementary file 7 — Supplementary Figure 5 [file 41398_2024_2904_MOESM7_ESM.tif]
